# Auditory category knowledge in experts and novices

**DOI:** 10.3389/fnins.2014.00260

**Published:** 2014-08-22

**Authors:** Shannon L. M. Heald, Stephen C. Van Hedger, Howard C. Nusbaum

**Affiliations:** Department of Psychology, The University of ChicagoChicago, IL, USA

**Keywords:** categorization, expertise, audition, distributional learning

## Abstract

What do listeners know about sounds that have a systematic organization? Research suggests that listeners store absolute pitch information as part of their representations for specific auditory experiences. It is unclear however, if such knowledge is abstracted beyond these experiences. In two studies we examined this question via a tone adjustment task in which listeners heard one of several target tones to be matched by adjusting the frequency of a subsequent starting tone. In the first experiment listeners estimated tones from one of three distributions differing in frequency range. The effect of tone matching in the three different distributions was then modeled using randomly generated data (RGD) to ascertain the degree to which individuals' estimates are affected by generalized note knowledge. Results showed that while listeners' estimates were similar to the RGD, indicating a central tendency effect reflective of the target tone distribution, listeners were more accurate than the RGD indicating that their estimates were affected by generalized note knowledge. The second experiment tested three groups of listeners who vary in the nature of their note knowledge. Specifically, absolute pitch (AP) possessors, non-AP listeners matched in musical expertise (ME), and non-AP musical novices (MN) adjusted tones from a micro-scale that included only two in-tune notes (B4 and C5). While tone estimates for all groups showed a central tendency effect reflective of the target tone distribution, each groups' estimates were more accurate than the RGD, indicating all listeners' estimates were guided by generalized note knowledge. Further, there was evidence that explicit note knowledge additionally influenced AP possessors' tone estimates, as tones closer to C5 had less error. Results indicate that everyday listeners possess generalized note knowledge that influences the perception of isolated tones and that this effect is made more evident with additional musical experience.

## Introduction

Category knowledge is essential for making sense of our complex auditory environments. From segmenting a speech stream into meaningful units to anticipating the resolution in a musical piece, auditory categories shape our understanding and enjoyment of acoustic events.

Generally speaking, there are two broad classes of category knowledge that can be applied to auditory objects that are critical to deriving meaning from our auditory environments. The first type of conceptual knowledge, which has been referred to as an isolated concept (Goldstone, 1996), stems from a direct, associative link to an acoustic event (e.g., gun shot or dog bark). For this reason, isolated concepts are grounded in specific non-symbolic perceptual experiences (cf. Barsalou, 1993, 1999). In such cases, heard acoustic patterns may be recognized by comparison to mentally stored templates or features. For example, recognition theories that posit simple comparison of a signal against a stored prototype or exemplars of a particular category representation do so without consideration of the relationship among categories or category representations. As such, the neural instantiation of an isolated concept can be thought to be similar to the classical notion of a feature detector, whether represented as an individual cell or as population responses. Previous research has shown that there are single neurons that appear to be selective for highly complex stimuli such as faces and shapes that are object-specific although generalized over some stimulus properties (e.g., Hubel and Wiesel, 1968; Bruce et al., 1981). In these kinds of theories, the response of a feature detector need not be influenced by the states of other related feature detectors; it simply becomes activated when the trigger features are physically present. In support of this view, Freedman et al. (2003) have found evidence that neuron responses for stimulus patterns corresponding to higher-level object categories in monkey IT are feature-based and appear invariant and unaffected by the category structure to which they belong. Although, there is evidence that PFC neurons encode a variety of abstracted information including perceptual categories (Freedman et al., 2001), attentional sets (Mansouri et al., 2006), numbers (Nieder et al., 2002), and behavioral schemas (Genovesio et al., 2005).

The second class of conceptual objects is referred to as interrelated concepts (Saussure, 1959/1916; Goldstone, 1996) and when applied to auditory objects systematically relates sound patterns to meanings in a web of knowledge, such that concepts are not solely defined in terms of their content or extensional mapping but also in terms of their relationship with other concepts in the system. Some theories posit that speech and music are understood largely due to their interrelated or systematic conceptual structure (Collins and Quillian, 1969; Lakoff, 1987; Potts et al., 1989). For interrelated concepts, other concepts within the system affect the intension (internally represented meaning relationships) of a given concept (Johnson-Laird, 1983). Moreover, the systematicity between related concepts allows for generalization using intensionality beyond similarity (e.g., Martin and Billman, 1994). Thus the difference in these classes of concepts depends on the systematicity of the interrelationships within the set of concepts. Isolated concepts lack this systematicity and therefore auditory objects that are not linked systematically should have little perceptual effect on each other.

For interrelated concepts, systematicity can be thought of as providing a virtual context that could influence the perceptual experience of auditory objects. Previous research has shown that although the general population does not possess the ability to label tones without the aid of a reference tone, they do demonstrate some sensitivity to the correct tuning of familiar music based on their long-term experience with music. For example, individuals tend to hum or sing songs at or near the original key in which they heard them (Levitin, 1994; Bergeson and Trehub, 2002). Individuals are also able to determine above chance if a familiar song is transposed one or two semitones (Terhardt and Ward, 1982; Terhardt and Seewann, 1983; Schellenberg and Trehub, 2003). Additionally, Smith and Schmuckler (2008) have demonstrated that non-musicians without absolute pitch performed better than chance at determining if an exceedingly familiar dial tone had been pitch shifted or not. These studies suggest that at least to some extent individuals store absolute pitch information as part of their detailed representations of specific auditory experiences, such as a frequently heard melody or dial tone. It is unclear, however, if this pitch information is abstracted from these specific experiences to form a categorical representation for generalized note knowledge in long-term memory (Posner and Keele, 1968; Goodman, 1972; Reed, 1972; Barsalou, 1983; Murphy and Medin, 1985). If this the case, then effects of such knowledge should be seen on stimuli that the listener has not heard before, such as isolated sinewave tones. More specifically, if the sensory trace for a given tone is disrupted due to backward masking, individuals would have to rely on category level knowledge in order correctly estimate the tone. As such, the error in people's estimates can be used to reveal the nature of underlying category information.

The current set of studies thus aims to explore the nature of isolated pitch perception and the degree to which it is guided by generalized note knowledge by using a tone adjustment task in which listeners hear one of several target tones backward masked by white noise followed by a starting tone. In the task, listeners were asked to adjust a starting tone's pitch to match the target tone's pitch. Because the target tone was backward masked by white noise, individuals had to rely on category knowledge in order to correctly estimate the tone given that the sensory memory for the target (or the echoic memory) was no longer available (Massaro, 1975). In order to determine if listeners' estimates are affected by generalized note knowledge, we asked listeners' to estimate tones from one of three different acoustic frequency distributions of target tones, which were all tones from the Western scale. If listeners do not possess generalized note knowledge to guide their estimations, their responses should be based solely on the local context and stimulus properties to the extent these are available after masking, for a given target such that their estimates are no different than randomly generated data (RGD). Randomly generated data can be produced by simulating responses drawn randomly from the set of possible frequency responses available on any given trial. The arbitrary responses are simply created by using a random number generator to select a value that corresponds to a tone within the stimulus distribution for any condition. This arbitrary response can then be subtracted from the true target tone location, similar to how response error is found for real participants. To adequately model random responses it is necessary to match the number of simulated subjects for each distribution to the number of participants for each distributional set. These randomly generated responses represent a model that assumes that a listener has no access to the representation of the actual target tone pitch given that it was masked, but represent the starting tone pitch and then generate random responses from that point irrespective of the target tone frequency. To the extent that the RGD models participant responses successfully, it suggests that listeners maintain no abstract representation of the target. To the extent that participant responses deviate from the model in terms of improved performance, this demonstrates the formation of an abstract representation of the target tone even after masking. Prior experiments suggest that individuals possess some degree of absolute pitch information for specific auditory experiences (Terhardt and Ward, 1982; Terhardt and Seewann, 1983; Levitin, 1994; Bergeson and Trehub, 2002; Schellenberg and Trehub, 2003). If this information is also abstracted from these experiences in the form of generalized note knowledge that can sufficiently impact isolated tone estimates, then we should find significant differences between listeners' tone estimates and the RGD.

The second experiment builds upon the results from the first study by examining the effect musical training and possession of true absolute pitch may have on the estimation of tones. There are extreme individual differences found in the population with regard to auditory expertise. In fact, the extreme individual differences in auditory expertise within the auditory domain, makes it particularly well suited to examine the impact of long-term prior knowledge in perception. For instance, a small portion of the population possesses absolute pitch (AP)—the ability to correctly identify an isolated musical note without the aid of a reference note. Any listener with absolute pitch presents an idealized case of a listener who should be unaffected by masking given that such listeners can immediately recode the fragile auditory target representation into a stable note category. Thus such listeners, by comparison to the RGD, present a standard of classification of isolated pitches based on the intensional structure of music rather than simple frequency-pitch auditory mapping. Additionally, individuals widely vary in the amount of musical training they receive. Musical training has been shown to be related to improvements in auditory and visual working memory (Chan et al., 1998; Brandler and Rammsayer, 2003; Ho et al., 2003; Jakobson et al., 2003, 2008; Zafranas, 2004) as well as enhancements in attentional control (Hannon and Trainor, 2007). As such, the second experiment was designed to examine the differences in tone estimation for three different groups of listeners—absolute pitch (AP) possessors, non-AP individuals with matched musical expertise (ME), and non-AP musical novices (MN) on a micro-scale distribution where test tones differed by 20 cents and included two perceptually in tune notes (B4 and C5). Any differences in tone estimation found between AP listeners and ME should largely be due to explicit absolute pitch knowledge, while any differences between MN and ME should largely be due to music theoretic and music practice expertise.

## Experiment 1

**Figure 1:**
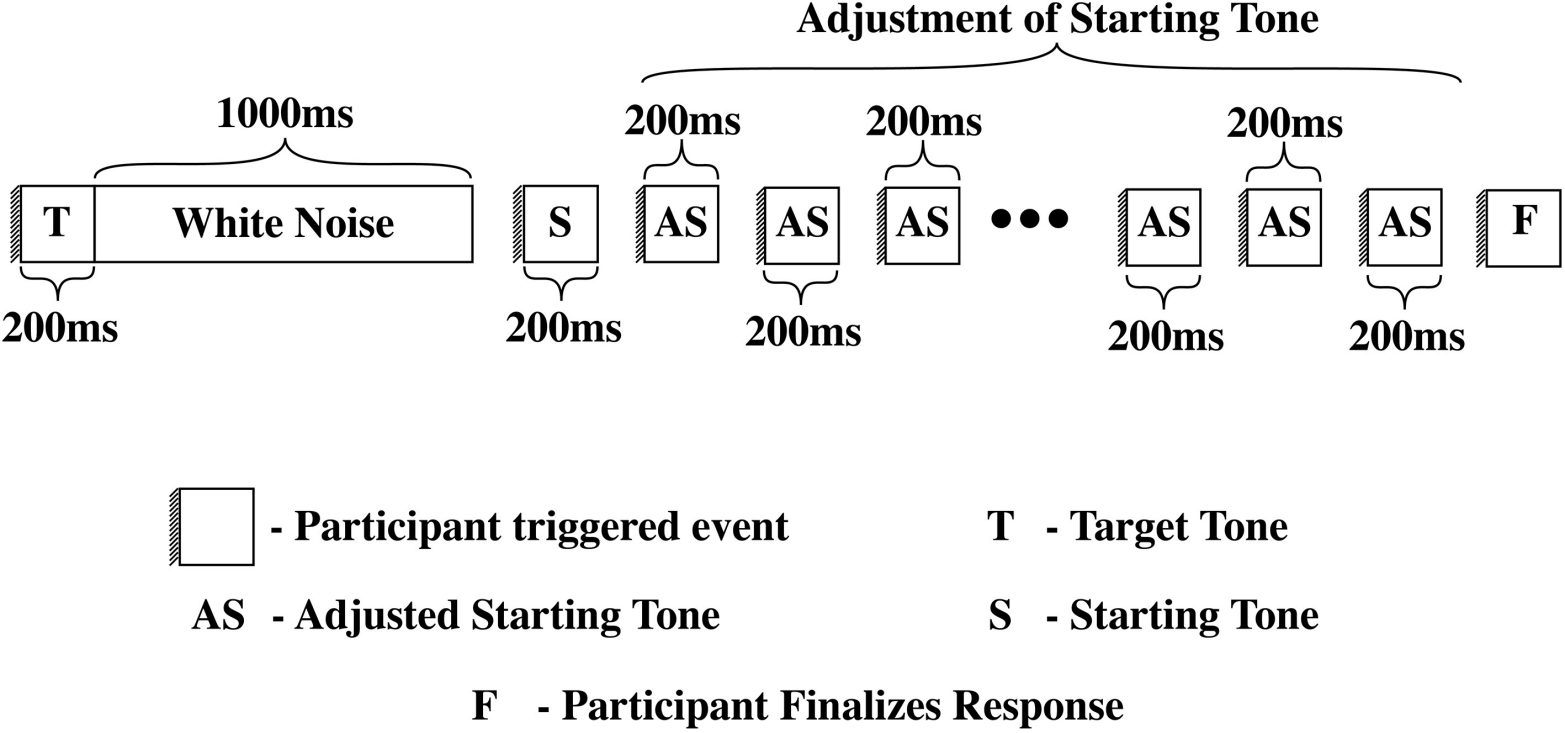
**On each trial, participants heard the target tone by clicking a circle with a T on it**. This tone lasted 200 ms and was followed by 1000 ms of white noise. Individuals then heard the starting tone by clicking a circle with an S on it. There was no time limit between when the subjects heard the target tone to when they clicked the circle with an S on it to hear a starting tone. Individuals were then asked to adjust the starting tone to match the target tone. Participants adjusted the starting tone, traversing the stimulus series, either in 66-cent increments or 33-cent increments. Individuals were given as much time as they needed to adjust the starting tone. When participants were satisfied with their adjustment, they confirmed their answer by pressing a circle with a C on it.

**Figure 2:**
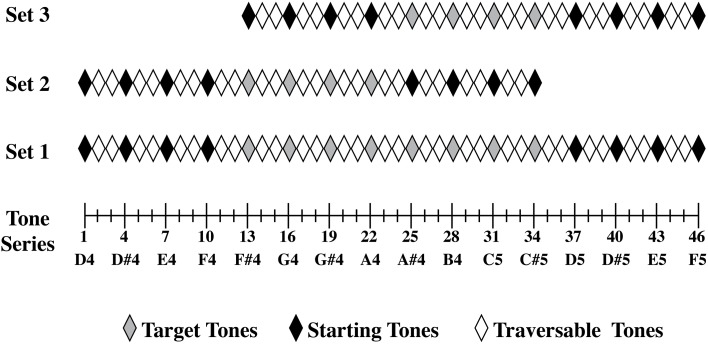
**Three sets of tones (or distributions of tones) were constructed from the original pure tone series that ranged from [D4] to [F5]**. Set 1 consisted of stimuli 1–46 from our pure tone series, set 2 consisted of stimuli 1–34 from our pure tone series, and set 3 consisted of stimuli 13–46 from our pure tone series. In set 1, stimuli 1, 4, 7, 10, 37, 40, 43, and 46 were used as starting tones, while stimuli 13, 16, 19, 22, 25, 28, 31, and 34 were used as target tones. In set 2, stimuli 1, 4, 7, 10, 25, 28, 31, and 34 were used as starting tones, and stimuli 13, 16, 19, and 22 were used as target tones. In set 3, stimuli 13, 16, 19, 22, 37, 40, 43, and 46 were used as starting tones and stimuli 25, 28, 31, and 37 were used as target tones.

In order to examine the degree to which long-term knowledge influences the perception of isolated tones the present experiment used a tone adjustment task in which a target tone was presented at a specific frequency and then backward-masked with white noise. Backward-masking was used to reduce the availability of the sensory trace (or echoic memory) of the tone (Massaro, 1975) and instead rely on more abstract category level knowledge. Following the target tone and mask, listeners then heard a starting tone, which they were asked to adjust in frequency to match the pitch of the target tone (See Figure 1). Depending on the condition to which they were assigned, listeners were given target tones from one of three different distributions of the acoustic frequency of the tone stimuli (See Figure 2). The distributions of stimuli were constructed such that two distributions (Set 2 and 3) were a subset of the frequency range of the third distribution (Set 1). The manipulation of the frequency of the test sets was manipulated specifically to test for range effects in the tone matching judgments. For each trial the error or difference between listener's response and the actual test tone was measured. If listeners adjusted the starting tone, such that it was identical to the target tone, then was no error as the estimate was accurate. There were two kinds of errors that listeners could make; they could either over estimate or under estimate the target frequency. The error between the adjusted tone and the target tone was measured in 33-cent steps, as that was the smallest step size by which participants could traverse the distributions.

As previously mentioned, listeners were given target tones from one of three different distributions of stimuli. If listeners' specific auditory experiences are not abstracted in the form of generalized note knowledge, their responses should be based solely on the local acoustic frequency range context and the stimulus properties of the given stimulus tones. We modeled these effects using RGD, which showed that truly random frequency estimates would reflect the distribution of tested target tones. More specifically, random frequency estimates for lower pitched targets of a particular frequency distribution should on average show frequency over estimation, given that the probability of randomly selecting a tone higher than the target is greater than randomly selecting a probe response lower than the target due to frequency range limitations on the responses. Similarly, a random distribution of frequency estimates for higher pitched targets of a specific frequency distribution should on average show frequency underestimation, given that the probability of randomly selecting a response tone lower in frequency than the target is greater than randomly selecting a response probe tone frequency higher than the target. The most central members of a distribution should on average show zero error, as there would be an equal probability of randomly selecting a response probe tone that is either higher or lower in frequency than the target. By extension, stimulus sets with a frequency range that is larger should on average have greater overall error than stimulus sets with more restricted frequency ranges. The degree to which listeners' estimates reflect this response error pattern suggests the degree to which listeners' estimates are consistent with a random response model governed primarily by local stimulus context and target tone frequency. By contrast, if individuals possess musical note knowledge—in other words can identify the note category of a particular target tone–based on long term listening experience (Terhardt and Ward, 1982; Terhardt and Seewann, 1983; Levitin, 1994; Bergeson and Trehub, 2002; Schellenberg and Trehub, 2003) and this knowledge affects the frequency estimates of simple isolated target tones then we should find significant differences between listeners' tone estimates and the randomly generated responses.

### Methods

#### Subjects

Twenty nine undergraduates (18 male) were recruited from the University of Chicago undergraduate community and were between 18 and 26 years of age 1. While participants were not specifically recruited for their musical background, individuals reported to have studied or played an instrument (piano, violin, bass, guitar, flute, or singing) on average 5.5 years (*SD*: 4 years; Set1—M:3.6 years, *SD*: 3.3 years; Set2—M:6.3 years, *SD*: 4.7 years; Set3—M:6.5 years, *SD*: 4.2 years). Participants were either granted course credit or paid for their participation in the experiment. All participants had no reported history of either a speech or a hearing disorder. Additionally, informed consent, using a form approved by the University of Chicago Institutional Review Board, was obtained from all subjects.

#### Stimuli

A pure sinewave tone series ranging from [D4] to [F5] was generated using Matlab. (See Figure 2 for the range of stimuli.) All stimuli were 200 ms in duration, were RMS normalized to 75 dB SPL, and had a sampling rate of 10 kHz with 16-bit samples. The lowest tone in the series, at the [D4] end of the series, had a frequency of 293.64 Hz. For each succeeding tone in the series, the frequency was increased by one third of a semitone or 33 cents. A step size of 33 cents was chosen as it is well above most listeners' thresholds for detecting pitch differences (e.g., Hyde and Peretz, 2004), while making the task challenging. The highest tone of the series, at the [F5] end of the series, the tone had a frequency of 698.39 Hz. The frequencies of the sine tones used were based on an equal tempered scale using tempered intervals. The masking noise was random Gaussian white noise and was generated in Matlab. Similar to the other stimuli, the white noise also was RMS normalized to 75 dB SPL and had a sampling rate of 10 kHz with 16-bit samples. The white noise sample however, was 1000 ms in duration.

#### Procedure

The experiment consisted of a tone adjustment task in which a target tone was backward-masked with white noise and then matched by varying the frequency of a starting tone. On each trial, participants were asked to click a circle with a T on it to hear a target tone, followed by one second of white noise. Individuals were then asked to click a circle with an S on it to hear a starting tone. There was no time limit between when the subjects heard the target tone to when they clicked the circle with an S on it to hear a starting tone allowing listeners to pace the experiment comfortably. Individuals were then asked to adjust the starting tone to match the target tone. Participants adjusted the starting tone, traversing the stimulus series, by clicking either big or small arrows located above and below the circle with an S on it. Arrows above the circle allowed participants to move higher in frequency in the series, increasing the starting tone's frequency, while the arrows below the circle allowed participants to move lower in frequency in the series, decreasing the starting tone's frequency. The larger arrows modified the starting tone in 66-cent increments, while the smaller arrows modified the starting tone in 33-cent increments. Participants were told to use the larger arrows to quickly move through the series and then to use the smaller arrows to make fine grain adjustments to their answer. Individuals were given as much time as they needed to adjust the starting tone. When participants were satisfied with their adjustment and believed it matched the original target tone, they pressed a circle with a C on it to confirm their answer. Participants were then asked to press Space bar to continue to the next trial. All key press responses were recorded. The experiment was conducted binaurally over sennheiser HD570 headphones. Figure 1 depicts the event structure for a given trial.

Participants were assigned to one of three stimulus distributions that varied the acoustic frequency range in the presentation of both the starting tones and target tones. Range variation was manipulated to determine the degree to which tone estimates were random as the current task was constructed so that the more variable individuals' estimates were, the more their estimates would reflect the distribution of tested target tones. Set 1 consisted of tones 1–46 from the pure tone series, set 2 consisted of stimuli 1–34 from the pure tone series, and set 3 consisted of stimuli 13–46 from our pure tone series. Set 2 and 3 are different subsets of Set 1 shifted in frequency range.

Figure 2 depicts how each distributional set was constructed and which tones were used as starting tones and target tones. All starting tones and target tones in each set were actual notes in the Western music 12-note chromatic scale. Each starting tone and target tone combination was presented two times each. Multiple starting tones were used across trials and counterbalanced in a pseudorandomized order to remove any general over- or underestimation of the tones due to the starting tone's position. Set 1 had 8 target tones and 8 starting tones, so there were 128 total trials. Set 2 and 3 each had 4 target tones and 8 starting tones, there were 64 total trials. 9 individuals were asked to adjust tones from set1, 9 individuals were asked to adjust tones from set 2, and 11 individuals were asked to adjust tones from set 3.

As RGD is distributionally specific, RGD was created for each distributional set. The arbitrary responses were created by using a random number generator to select a value that corresponded to a tone within the distribution (1–46 for the large distribution in Experiment 1, 1–34 for the smaller distributions in Experiment 1, and 1–27 for the micro distribution used in Experiment 2). We then subtracted this arbitrary response from the true target tone location, just as we did for the real participants. The number of simulated subjects for each distribution was matched to the number of participants for each distributional set. Therefore, 9 simulated random subjects were run for set 1, 9 simulated random subjects were run for set 2, and 11 simulated random subjects were run for set 3.

### Results

We calculated the frequency matching error for each adjustment trial by subtracting the actual target tone's stimulus number in the frequency series from the adjusted starting tone's stimulus number that was associated with the participant's confirmed response. The calculated adjustment error therefore represented the number of 33-cent steps by which an individual's adjustment was in error. Some of the adjustment errors were so extreme that it appeared that the participant entered an arbitrary response, by either failing to attend to the target tone on that given trial or by accidently confirming their adjusted response before they had made any adjustment. To find these outliers, we culled final responses that were greater or less than two standard deviations away from the average participant's response. This procedure was repeated for each test tone. Only 5.1% of responses for target tones from set 1, 6.4% of responses for target tones from set 2 and 5.1% of responses for target tones from set 3 were removed in this procedure.

The groups that corresponded to each of the three frequency ranges for targets did not significantly differ (alpha 0.05) in the amount of time that they took to adjust the starting tone [*F*_(2, 28)_ = 0.303, *p* = 0.742] to match the target tone. Participants who adjusted tones from Distribution 1 took an average of 6 s (*SD*: 2.5 s); participants who adjusted tones from Distribution 2 took an average of 5.6 s (*SD*: 0.7 s); participants who adjusted tones from Distribution 3 took an average of 6.4 s (*SD*: 3 s). Overall, individuals who adjusted tones from distribution 1 finished the task within 50–55 min, while individuals who adjusted tones from distribution 2 and 3 finished the task within 25 min, as distributions 2 and 3 had half as many trials.

In order to examine the impact of generalized note knowledge on the perceptual judgments of isolated tones the amount of matching error was found for each target tone, for each of the three sets of tones tested. If individuals used generalized note knowledge in the perception of tones then we would predict all target tones should have similar amount of error. This is not the case. Three separate One-Way repeated measure ANOVAs with Target Tone as the main factor was carried out for each distribution set (group of listeners) for the dependent measure of error. For each distribution, the effect of Target Tone was significant [Set 1, *F*_(7, 56)_ = 21.255, *p* < 0.0001; Set 2, *F*_(3, 24)_ = 17.168, *p* < 0.0001; Set 3, *F*_(3, 30)_ = 23.722, *p* < 0.0001] indicating that the amount of error for at least one test tone out of each series was significantly different.

**Figure 3:**
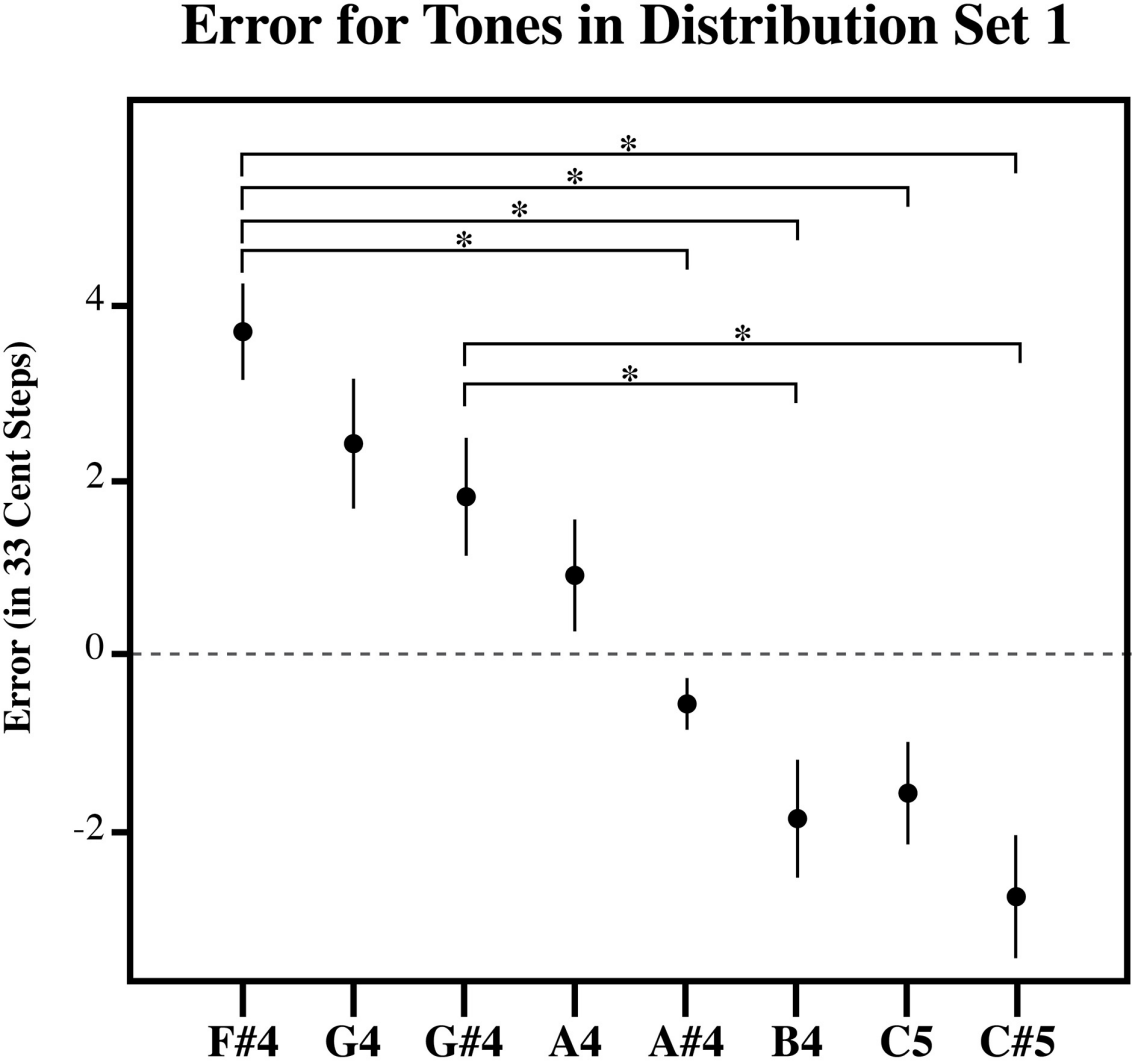
**The plot of the estimation error in 33-cent steps for each tone in Distribution Set 1**. Significant pairwise comparisons (alpha 0.05) using a Sidak adjustment are indicated with an asterisk. Note that while individuals bisected the presented distribution at its midpoint, such that lower target tones in the series are overestimated and higher target tones in the series are underestimated similar to the randomly generated tone estimate data, individuals produced estimates that were significantly more accurate than the RGD (Figure 6A). Error bars represent ±1 s.e.m.

**Figure 4:**
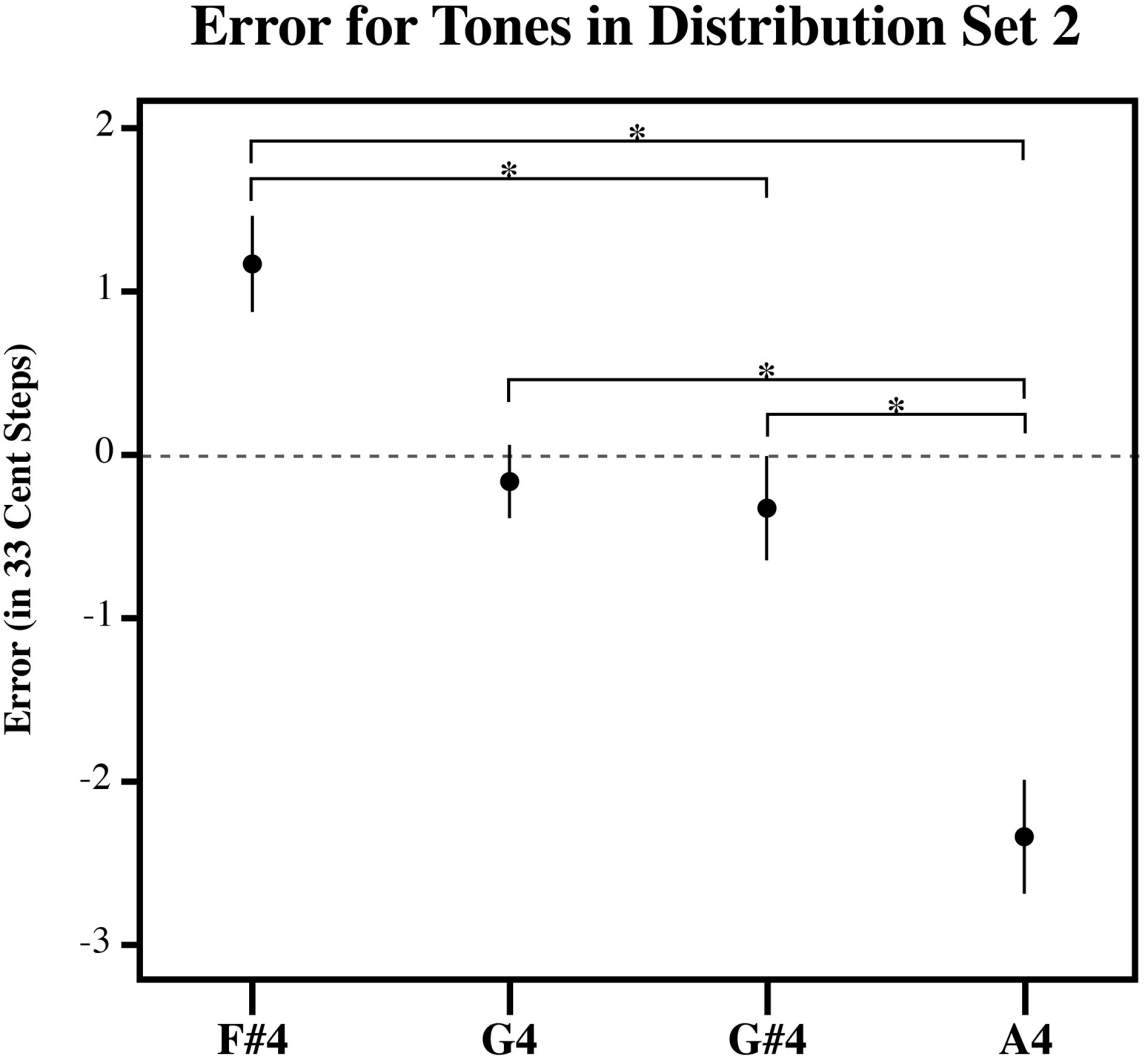
**The plot of the estimation error in 33-cent steps for each tone in Distribution Set 2**. Significant pairwise comparisons (alpha 0.05) using a Sidak adjustment are indicated with an asterisk. Note that while individuals bisected the presented distribution at its midpoint, such that lower target tones in the series are overestimated and higher target tones in the series are underestimated similar to the randomly generated tone estimate data, individuals produced estimates that were significantly more accurate than the RGD (Figure 6B). Error bars represent ±1 s.e.m.

**Figure 5:**
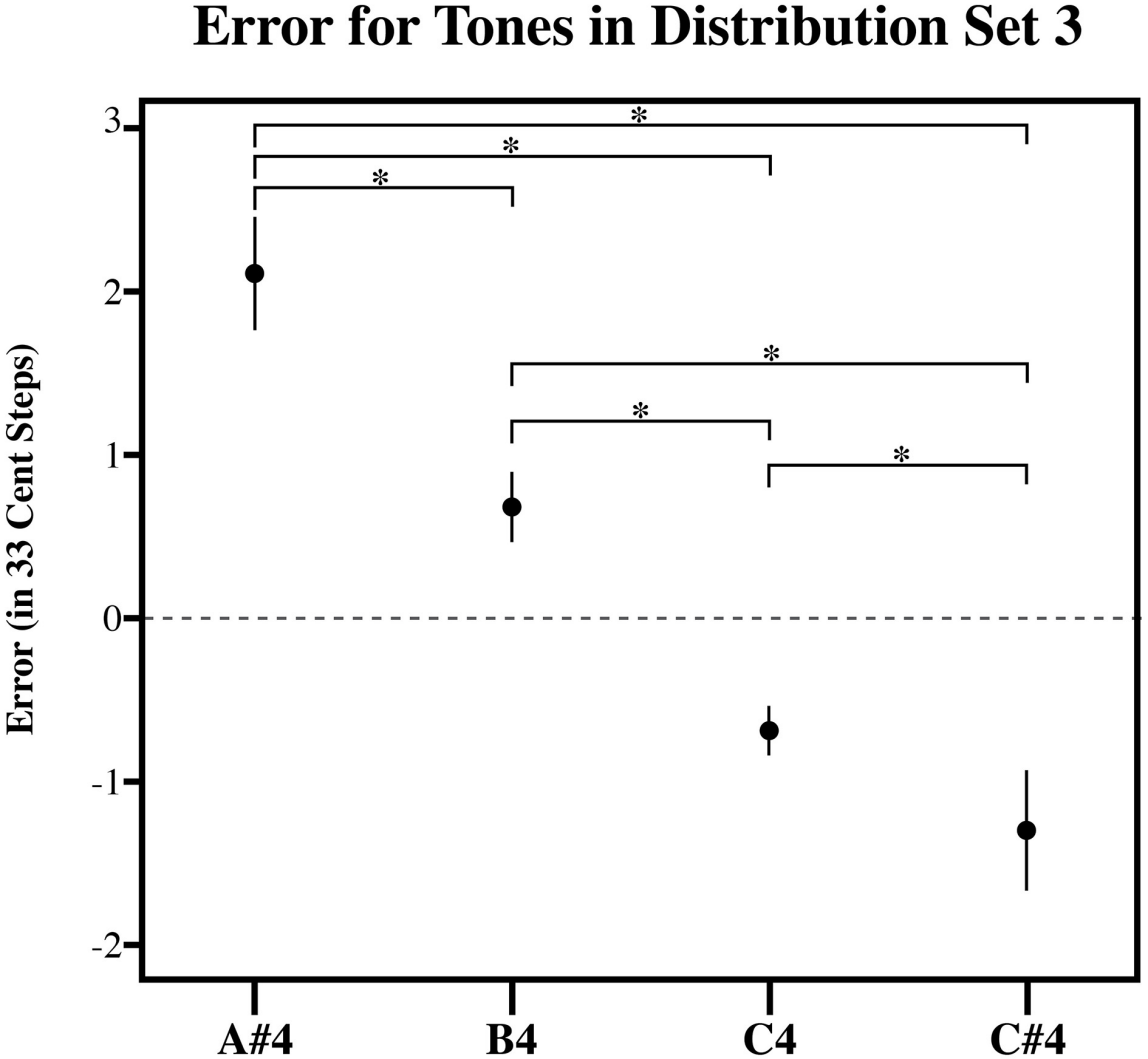
**The plot of the estimation error in 33-cent steps for each tone in Distribution Set 3**. Significant pairwise comparisons (alpha 0.05) using a Sidak adjustment are indicated with an asterisk. Note that while individuals bisected the presented distribution at its midpoint, such that lower target tones in the series are overestimated and higher target tones in the series are underestimated similar to the randomly generated tone estimate data, individuals produced estimates that were significantly more accurate than the RGD (Figure 6C). Error bars represent ±1 s.e.m.

**Figure 6:**
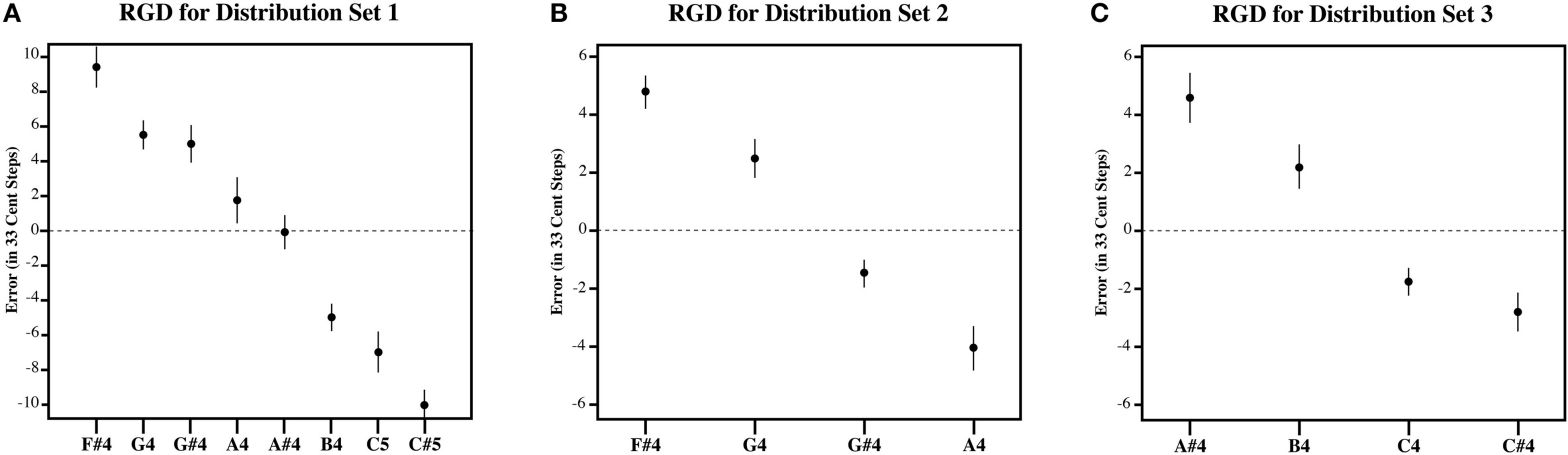
**The plot of the estimation error in 33-cent steps for each tone for the RGD**. **(A)** Shows the RGD for set 1, **(B)** shows the RGD for set 2, and **(C)** shows the RGD for set 3. Note that the RGD bisects the presented distribution at its midpoint, such that lower target tones in the series are overestimated and higher target tones in the series are underestimated. Error bars represent ±1 s.e.m.

Figures 3–5, plot the mean amount of error in 33-cent steps for each of the target tones for each of three distribution sets. Pairwise comparisons among the estimated marginal means were also performed using a Sidak adjustment. The significant (alpha 0.05) pairwise comparisons from these analyses are additionally shown in these figures. The RGD for each distribution set is additionally shown for comparison purposes in Figure 6. For the RGD data from set 1, all tones were significantly different from one another (using a Sidak adjustment—alpha 0.05) except tone 1 with 2, 3, and 4; tone 2 with 3 and 4; tone 3 with 4 and 5; tone 4 with 5, 7 and 8; tone 5 with 6 and 7; tone 6 with 7, and tone 7 with 8. In set 2 all tones in the RGD were significantly different from one another (using a Sidak adjustment—alpha 0.05) except tone 1 with 2; and tone 3 and 4. In set 3 all tones in the RGD were significantly different from one another (using a Sidak adjustment—alpha 0.05) except tone 1 with 2; and tone 3 with 4.

Visual inspection of the data shows that individuals' estimates were highly influenced by the distribution they received. For each distribution, higher pitched items showed underestimation as the probability of randomly selecting a tone lower than it was greater than randomly selecting a tone higher than it, while lower pitched items showed overestimation as the probability of randomly selecting a tone higher than it was greater than randomly selecting a tone lower than it, and central items showed near zero error as the probability of randomly selecting a tone both higher and lower than it was similar. In order to compare individual's estimates to the RGD, an error function was found for each participant (and each simulated subject in the RGD) by plotting the amount of error against the presented test tone series. Because the amount of error, when plotted against the target tone distribution used for each subject was linear in nature, a linear regression line was then fitted to each error function. From this, the x-intercept was calculated to infer the point of zero error. For distributional set 1 (the superset), the point of zero error was between stimulus 23 and 24, at 23.64. This point mirrors closely the actual midpoint of distributional set 1, which is between stimulus 23 and 24. For distributional set 2 (the lower frequency range), the point of zero error was between stimulus 16 and 17 at 16.86. Again, this is very similar to the actual midpoint of distributional set 2, which is between stimulus 17 and 18. For distributional set 3 (the higher frequency range), the point of zero error was at stimulus 30 at 30.00. This also closely echoed the true midpoint of distributional set 3, which is between stimulus 29 and 30. This indicates that individuals were highly sensitive to the presented distributions, suggesting that individuals' estimates were variable in nature.

While, individuals' general pattern of estimation error across the tones reflected a pattern consistent with context sensitive range-dependent estimates, listeners' responses may significantly and meaningfully differ in other ways from the RGD. For example, it is possible that while individuals' estimates were inexact, individuals did use their experience with Western music to inform their estimates. If this is the case, individuals should display significantly less absolute error across the distribution than found in the randomly generated estimates. To test this, the amount of estimation error was plotted against the presented test tone series for each subject. A linear regression line was fitted to each subject's estimation error function. This was also done for the RGD. The steepness of the fitted linear regression line was then used to assess the degree to which items were judged with more error, a steeper fitted regression line would necessarily denote more extreme overestimation of smaller items as well as more extreme underestimation of larger items in the series. As such, the slope corresponding to the fitted regression lines was used as a dependent measure in three separate planned independent sample *T*-tests (equal variances no assumed) to examine if individuals from each of the three distributions significantly differed from the RGD. Indeed, for each of the three distributions, individuals' responses showed significantly less error than the RGD [Set 1: *t*_(14.6)_ = 7.456, *p* < 0.001; Set 2: *t*_(11.7)_ = 7.46, *p* < 0.001; Set 3: *t*_(16.3)_ = 3.88, *p* = 0.001]. This suggests that individuals possess a limited amount of long-term pitch knowledge that helped to constrain their target tone pitch matching estimates.

### Discussion

The present results are similar to previous findings that suggest that individuals in the general population have some generalized absolute pitch knowledge (Terhardt and Ward, 1982; Terhardt and Seewann, 1983; Levitin, 1994; Bergeson and Trehub, 2002; Schellenberg and Trehub, 2003). Individuals' estimates of the frequency of isolated target notes are guided to some extent by generalized note knowledge. For each tested distribution, individuals were significantly more accurate than the RGD. This suggests that listeners used abstracted note information that goes beyond the specific auditory context from which they were experienced, to form generalized note knowledge. However, while we found some evidence that long-term pitch knowledge helped to make estimates more accurate, individuals' estimates were still highly inaccurate. Pitch matching error for any given tone was largely dependent on the stimulus range in which it was presented. Individuals' pitch matching estimates were significantly influenced by the distribution of possible tones as they overestimated the lower pitched tones and underestimated the higher pitched tones in each distribution. Moreover, their estimates were most exact for the center of each tested distribution. Strikingly, this point of zero error directly mirrored the actual midpoint of each series. This sensitivity to the distribution is consistent with the idea that while individuals possess some generalized note knowledge, their estimates of isolated target notes are still variable in nature.

There are several possibilities as to why we failed to find strong evidence for latent note knowledge in the frequency estimates of target tones. One possibility is that prior pitch knowledge for individuals in the general population may be representationally sparse or underspecified due to poor encoding or insufficient experience. Another possibility is that individuals may not have recognized the notes in this experiment as examples of musical tones as they were pure sine wave tones. As such, they simply did not bring their latent note knowledge to bear on their estimates of these tones, since sine wave tones do not generally occur in every day musical experience.

While the general population does not have the ability to name a note without a reference note as guide, listeners with absolute pitch (AP) can do so and have well defined note categories. As such, this knowledge should affect their estimates of isolated tones. When people make use of category level knowledge, the most typical or central members of a category are best remembered, whereas items less typical or more extreme are distorted by this knowledge and are perceptually judged as being more typical or less extreme than they actually are. This effect is known as a central tendency effect. As such, Individuals with AP should make estimates that are influenced by their long-term absolute category knowledge. In this sense, the most typical members (perfectly tuned notes) should exhibit zero error, whereas mistuned notes within the category should be distorted by their category knowledge and be remembered as more typical then they actually are. The next experiment was conducted to further understand how such prior knowledge influences the estimation of isolated tones.

## Experiment 2

In order to better understand how prior note knowledge affects the pitch estimates for isolated tones, Experiment 2 investigated whether differences in prior chromatic scale experience moderates the results reported in Experiment 1. In the present experiment, a fixed frequency range of stimuli was used with only two correctly tuned notes and 12 tones that were mistuned from those notes as target tones. The two correctly tuned notes were on either side of the center of the distribution so that prior pitch knowledge would be juxtaposed against a random or highly variable estimation pattern. To the extent that individual judgments are random, individuals' general pattern of estimation error across the tones should reflect a pattern consistent with context sensitive, range-dependent estimates. This is the case, as a model of randomly generated responses indicates that random estimates for lower frequency target tones of a distribution should on average be over estimated as the probability of randomly selecting a tone higher than it is greater than randomly selecting a tone lower than it, while random estimates for higher frequency items of a distribution should on average be underestimated as the probability of randomly selecting a tone lower than it is greater than randomly selecting a tone higher than it. Most importantly, even if individuals' estimates are random, there should be no estimation error for judgments at the center of the stimulus series despite the fact that the center is not actually a correctly tuned note. However, if prior note knowledge is abstracted to form generalized note knowledge that helps to inform individuals' pitch estimates for target tones, the two notes that are correctly tuned in the stimulus series should show reduced error. This is because the most typical or central members of a category should be best remembered, whereas items less typical or more extreme are distorted by this knowledge and are perceptually judged as being more typical or less extreme than they actually are. For this reason, listeners with absolute pitch (AP) should demonstrate less variability in their estimates. Thus, it is possible that individuals with more specific note knowledge (either explicitly in the for of AP knowledge, or more generally as more musical experience) will not show zero error for the mistuned center of the stimulus series. Instead, these individuals may show zero error for the targets that are the two correctly tuned notes. Further, neighboring tones that are slightly sharper than these in-tune tones should be underestimated, while tones that are slightly flatter than these in-tune tones should be overestimated.

However, it is also possible that AP listener judgments of notes will still show some pitch estimate error despite their note knowledge. A study by Hedger et al. (2013) provides clear evidence that AP perception is dependent on the tuning of recent experiences with particular notes and timbres, and that it is not reliant upon a direct or naïve realism framework (cf. Gibson, 1972), in which the underlying note is directly perceived. Given that the context of recent musical experience is important in the maintenance of note categories for AP listeners, AP listeners may show some error in their pitch estimates of isolated tones.

While it is clear that AP listeners have more extensive prior perceptual note knowledge than non-AP listeners, it is possible that AP listeners differ in other meaningful ways. For example, AP listeners will on average have more extensive music experience compared to the general population. There is a large body of literature that suggests that musical training is correlated with domain-general enhancements in cognitive processing. For example, music training is positively correlated with performance in auditory and visual working memory tasks (Chan et al., 1998; Brandler and Rammsayer, 2003; Jakobson et al., 2003, 2008; Ho et al., 2003; Zafranas, 2004) as well as with improved attentional control (Hannon and Trainor, 2007). Better auditory working memory or attentional control from musical training could help AP listeners perform better in the tone matching task by improved memory for target notes.

Therefore, we compared performance in a tone adjustment task for AP listeners with two additional groups–musical experts (ME) and true musical novices (MN). Schlemmer (2009) suggests that that ME may have richer generalized musical note knowledge than MN, as evidenced by a positive correlation between musical expertise and the ability to spontaneously sing a well-rehearsed piece on key without the aid of a reference tone. Overall though, note judgment differences between AP listeners and ME should be due to absolute pitch, while performance differences between MN and ME should be largely due to music theoretic and music practice expertise.

### Participants

In order to understand how prior absolute pitch knowledge might influence the estimation of tones we recruited musical novices (MN), musical experts (ME) and Absolute Pitch listeners (AP) to take part in a tone-probe adjustment task similar to Experiment 1. Thirty-one individuals (11 musical experts, 4 females; 12 musical novices, 8 females; and 10 absolute pitch listeners, 6 females) participated in the experiment. The musical experts had studied or played an instrument (piano, violin, viola, cello, flute, singing) for at least 15 years (*M*: 23.1 years, *SD*: 7.7 years); all experts had training in the theory of harmony and in counterpoint during their studies. Musical novices had limited to no experience playing an instrument or singing (*M*: 2.9 years, *SD*: 3.2 years).

Absolute pitch listeners both identified themselves as possessing AP, but also passed a test that verified their ability to accurately produce isolated notes (for details, see the Procedure Section). Absolute pitch listeners, similar to musical experts reported substantial musical expertise, reporting to have studied or played an instrument (piano, violin, viola, cello, flute, singing) for at least 11 years (*M*: 22.1 years, *SD*: 9.9 years). All participants had no reported history of either a speech or a hearing disorder. Participants were either granted course credit or paid for their participation in the experiment.

Additionally, informed consent, using a form approved by the University of Chicago Institutional Review Board, was obtained from all subjects.

### Stimuli

A 27-stimulus, pure sinewave tone test series ranging from a frequency that was 20-cent sharp [B^b^4] to a 20-cent flat [C^#^5] was generated using Matlab. All stimuli were 200 ms in duration. For Stimulus 1, at the [Bb4] end of the series, the tone had a frequency of 471.58 Hz. For each succeeding stimulus in the series, the frequency was decreased by one tenth of a semitone or 10 cents. A step size of 10 cents was chosen as it is toward the lower end of the range of most listeners' thresholds for detecting pitch differences (e.g., Hyde and Peretz, 2004), helping to make the task challenging even for AP possessors and ME. Consequently, for Stimulus 27, at the 20-cent flat [C^#^5] end of the series, the tone had a frequency of 547.99 Hz. The frequencies of the sine tones used were based on an equal tempered scale using tempered intervals.

The use of a finer grained distribution than in Experiment 1 allowed us to pit prior category knowledge against context-sensitive responses. If individuals' estimates are influenced by prior absolute pitch knowledge, then central tendencies or points of zero error should be observed for stimuli 9 and 19, as these tones of the series are, by Western music standards, perfectly tuned notes (B4 and C4). Tones near these perfectly tuned notes should be affected by prior absolute pitch knowledge, causing them to be remembered as more typical then they actually are. This means that slightly sharp notes will be underestimated, while slightly flat notes will be overestimated. Conversely, to the extent that individuals' estimates are variable, a point of zero error should be observed at or near stimulus 14, as this is the center of the tested distribution.

### Procedure

The experiment consisted of two parts. First, the participants were introduced to the stimuli of the experiment via a grouping task. The sole purpose of this task was to make certain that individuals understood the tones to be examples of musical notes, as the stimulus series only contained two perfectly in-tune notes. The grouping task therefore ensured that AP possessors, MN, and ME would use whatever prior note knowledge they have in perception of the tones. The grouping task was not necessary in Experiment 1 as all starting tones and target tones used in that experiment were actual notes in the Western music 12-note chromatic scale. In the grouping task all 27 stimuli appeared as clickable and moveable objects (gray squares) on a computer screen. Each object was marked by a random 3-digit number as an arbitrary label. For each participant, the stimuli appeared in a random order at the top of the computer screen to avoid any presentation ordering effects. Before beginning the task the subjects were told that they would have the opportunity to listen to and organize a set of sine wave tones. To indicate that these were indeed examples of musical tones even though a majority of the tones were not in tune notes, participants were also informed that the tones they would hear were examples of Bb, B, C and C# notes, but that some tones would be better examples of these notes than others. Subjects were asked to first listen to each stimulus by clicking on each object. They were then asked to sort the tones into the previously mentioned groups: Bb, B, C and C#. During this portion of the task the participant could hear each stimulus as many times as they wished in order to group the tones appropriately. Each subject took approximately 15 min to complete this portion of the experiment.

**Figure 7:**
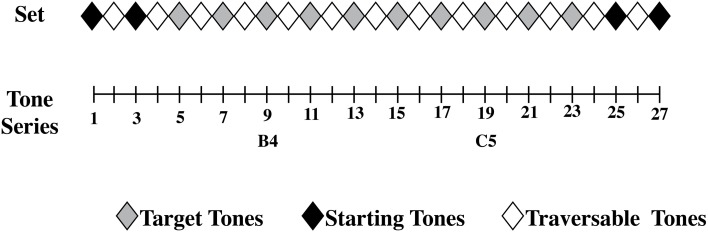
**The set of pure sinewave tones used for Experiment 2 ranged from a sharp [B4^b^] to flat [C5^#^]**. Stimuli 1, 3, 25, and 27 were used as starting tones, while stimuli 5, 7, 9, 11, 13, 15, 17, 19, 21, and 23 were used as test tones. Stimulus 9 was a perfectly in-tune [B4] and stimulus 19 was a perfectly in-tune [C5]. Stimulus 14, between stimuli 9 and 19, marks the central item of the tested distribution.

The second part of the experiment consisted of the tone adjustment task used in Experiment 1. The experiment was conducted binaurally over sennheiser HD570 headphones. Individuals adjusted tones from the set that they sorted in the grouping task. Figure 7 shows how the set of tones was constructed and which tones were used as target tones and starting tones. Participants experienced each starting tone and test tone combination two times each, for a total of 80 trials. Multiple starting tones were used across trials in order to counterbalance, and thus remove any general over- or under-estimation of the tones due to the starting tone's position.

After the grouping and adjustment task, AP possessors completed a test of their AP ability. In a sound-attenuating booth, AP possessors were asked to sing or hum isolated notes, the names of which appeared one-at-a-time on a computer screen. Black key notes were produced eight times each—four times with the sharp symbol (#), and four times with the flat symbol (b)—while white key notes were represented four times each. There were thus 58 total trials. Participants could produce the notes in any octave they wished, and were instructed to hold a steady note for at least 2 s in order to accurately analyze the pitch.

In order to determine if individuals' estimates were better than RGD, simulations of the tone adjustment task were accomplished for the distributional set. As RGD is distributionally specific, RGD was created for each distributional set. The arbitrary responses were created by using a random number generator to select a value that corresponded to a tone within the distribution. We then subtracted this arbitrary response from the true target tone location, just as we did for the real participants. A total of 12 simulated subjects were run.

### Results

The same scoring and culling methods used in Experiment 1 was used in Experiment 2. Outlier responses were removed at a rate of 3.6% of responses for test tones for APP, 3.3% of responses for test tones for ME, and 2.1% of responses for MN were removed in the culling procedure.

The three groups did not significantly differ (alpha 0.05) in the amount of time that they took to adjust the starting tone to the final response on average across trials [*F*_(2, 32)_ = 0.763, *p* = 0.475]. AP possessors took an average of 7.2 s (*SD*: 4.9 s), ME took an average of 5.7 s (*SD*: 1.5 s), and MN took an average of 5.7 s (*SD*: 2.4 s). Overall, individuals were able to complete the task within 30–35 min.

**Figure 8:**
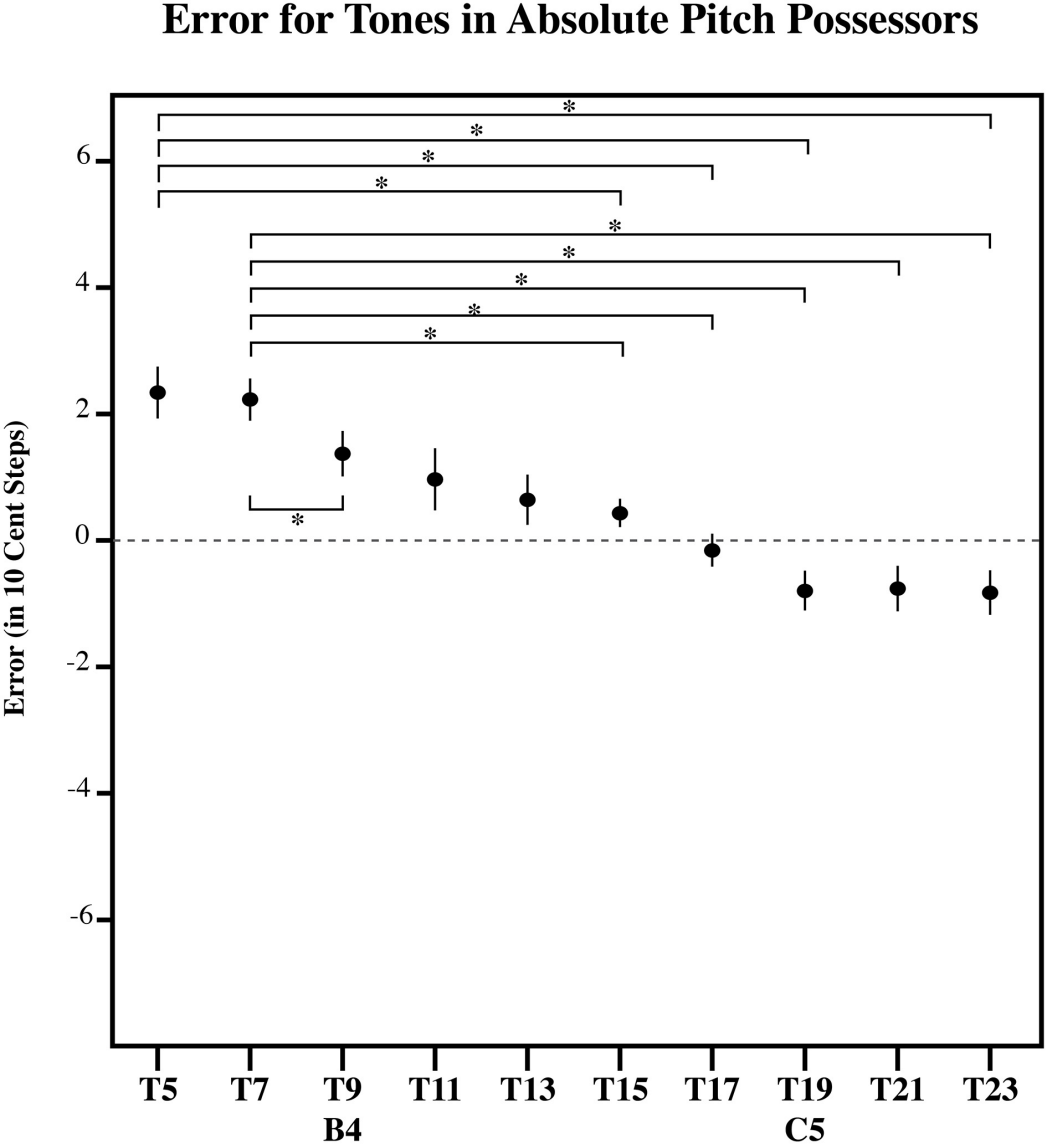
**The error in 10-cent steps for each tone in AP possessors**. Significant pairwise comparisons (alpha 0.05) using a Sidak adjustment are indicated with an asterisk. Note that AP possessors bisect the presented distribution at its midpoint, such that lower tones in the series are overestimated and higher tones in the series are underestimated. However, note that this point for is closer to [C5] in AP possessors, than in ME (Figure 9), MN (Figure 10), and the RGD (Figure 11). Additionally, AP possessors (Figure 8) and ME (Figure 9), have flatter error patterns than MN and the RGD (Figures 10, 11). Error bars represent ±1 s.e.m.

**Figure 9:**
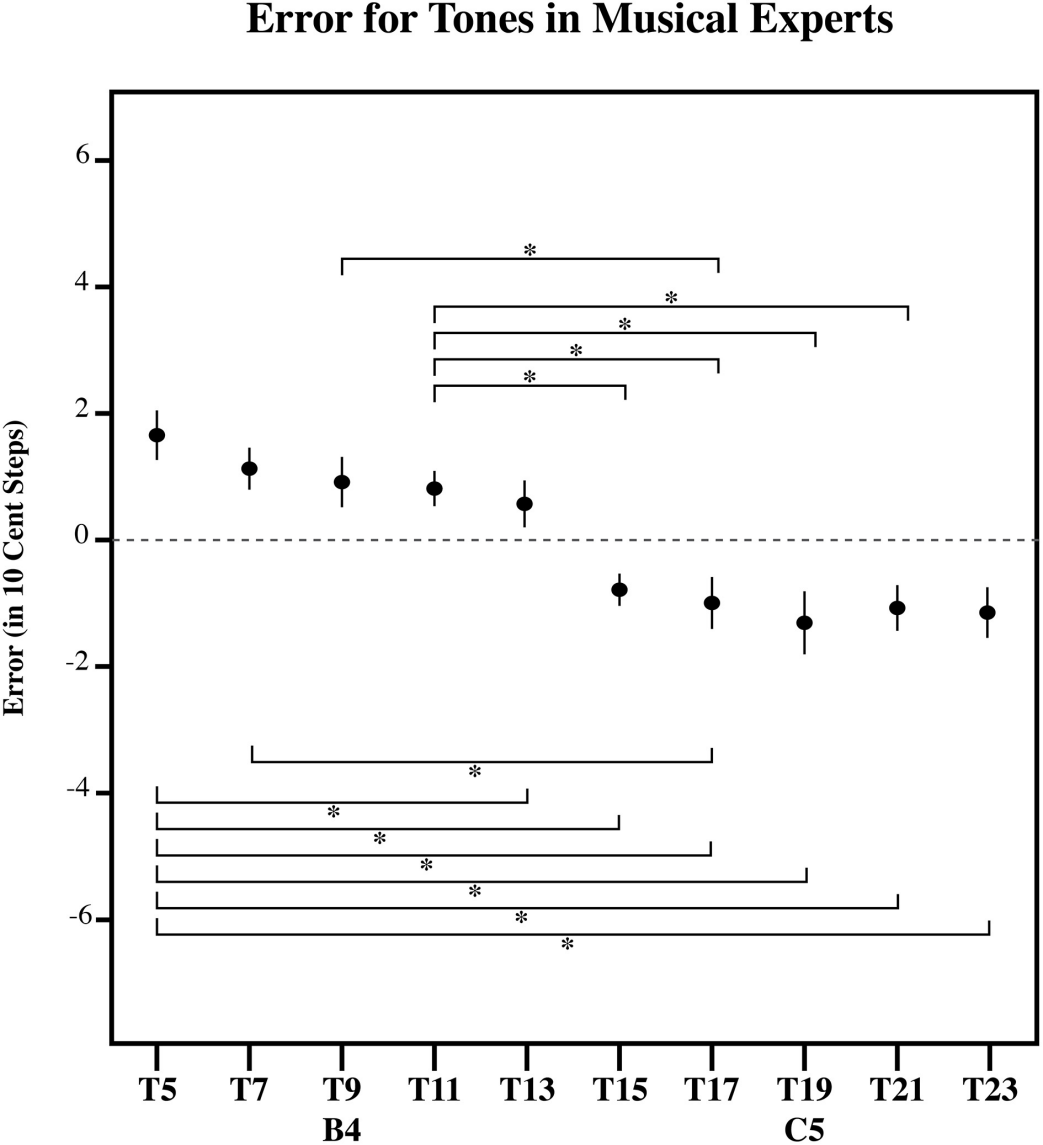
**The error in 10-cent steps for each tone in ME**. Significant pairwise comparisons (alpha 0.05) using a Sidak adjustment are indicated with an asterisk. Note that ME bisect the presented distribution at its midpoint, such that lower tones in the series are overestimated and higher tones in the series are underestimated. However, note that ME (Figure 9), along with AP possessors (Figure 8) have flatter error patterns than MN (Figure 10) and the RGD (Figure 11). Error bars represent ±1 s.e.m.

**Figure 10:**
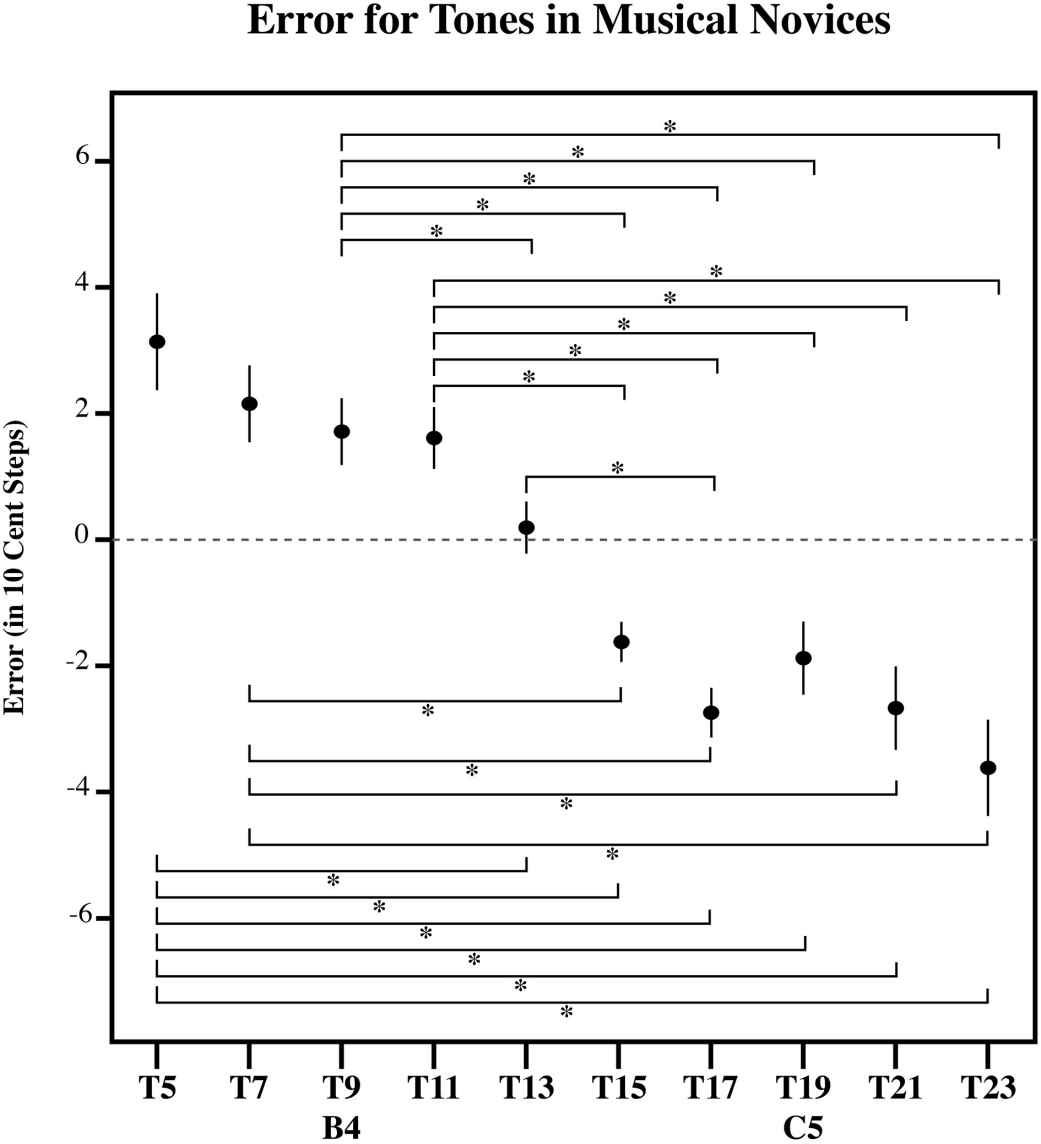
**The error in 10-cent steps for each tone in MN**. Significant pairwise comparisons (alpha 0.05) using a Sidak adjustment are indicated with an asterisk. Note that MN bisect the presented distribution at its midpoint, such that lower tones in the series are overestimated and higher tones in the series are underestimated. However, note that MN (Figure 10) has a flatter error patterns than the RGD (Figure 11). Error bars represent ±1 s.e.m.

As previously mentioned, the set of tones tested was specifically chosen to contrast prior category knowledge (two correctly tuned note targets) against the central tendency effect found in modeled RGD (the mistuned center of the stimulus series). If individuals' probe judgments are influenced by prior perceptual note knowledge, points of zero error should be observed for the two in-tune tones of the series (stimuli 9 and 19, B4 and C5. respectively). Additionally, neighboring out of tune stimulus tones that are slightly sharper than these in-tune tones should be underestimated while those tones that are slightly flatter than these in-tune tones should be overestimated. However, none of the groups' matching error responses reflected this pattern (see Figures 8–**10**). Instead, all three groups showed a point of zero matching error near the center of the tested distribution, which is not a correctly tuned note. This means that the lower pitched items of the test tone series showed positive error (or overestimation) and higher pitched items of the test tone series showed negative error (or underestimation). This suggests that all individuals' estimates were variable to some degree.

In order to determine if the amount of matching error for each test tone was different across the groups, we ran an omnibus repeated measures ANOVA with Target tone (10 different test tones were given as targets to match) as a repeated factor and Group (APP, ME, and MN) as a between subject factor. A significant main effect for Target tone was found [*F*_(9, 270)_ = 38.01, *p* < 0.001] indicating that the amount of error for at least one test tone out of the series was significantly different regardless of the listener group. Additionally, a significant main effect for Group was found [*F*_(2, 30)_ = 5.208, *p* < 0.01] denoting that musical experience or difference in prior pitch knowledge significantly altered the overall amount of matching error. Further, a significant interaction between Target tone and Group was found [*F*_(18, 270)_ = 3.398, *p* < 0.001] indicating that differences in musical experience or in prior note knowledge did not just globally lead to better or worse performance, but that they changed the judgments for each tone differentially.

**Figure 11:**
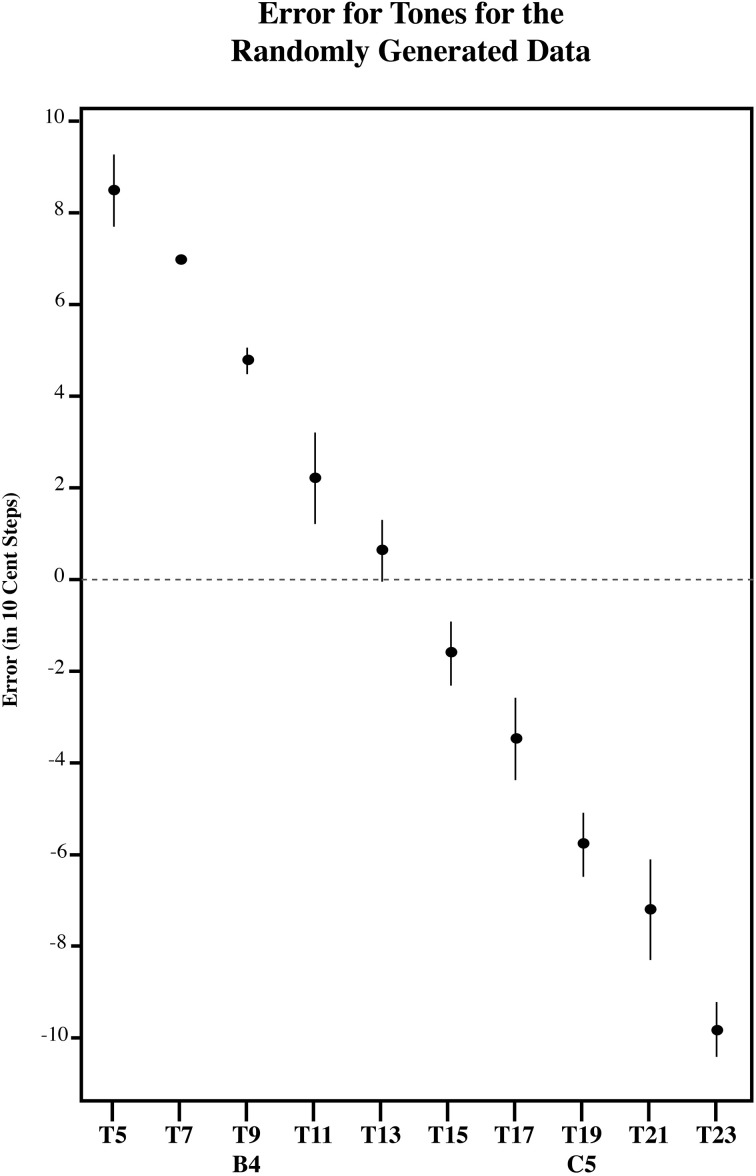
**The error in 10-cent steps for each tone in for the RGD**. All tones are significantly different from one another using a Sidak adjustment (alpha 0.05) except tone 1 with 2; 3 with 4; 4 with 3, 5, 6, and 7; 5 with 6, and 7; 6 with 7 and 8; 7 with 8 and 9; 8 with 9 and 10; and 9 with 10. Note that the RGD bisects the presented distribution at its midpoint, such that lower tones in the series are overestimated and higher tones in the series are underestimated. However, note that the RGD (Figure 11) has a steeper error pattern than AP (Figure 8), MN (Figure 10), and ME (Figure 9). Error bars represent ±1 s.e.m.

To further examine the effect of target tone for each listener group, we carried out three separate simple effects One-Way ANOVAs, one for each group (AP, MN, and ME). Each of these One-Way ANOVAs had a significant main effect for Target tone. [For AP, *F*_(9, 81)_ = 13.13, *p* < 0.001; for ME, *F*_(9, 90)_ = 11.91, *p* < 0.001; for MN, *F*_(9, 99)_ = 18.60, *p* < 0.001]. This suggests that for all three groups, at least one target tone out of the series was significantly different. Figures 8–10 plot the amount of error in 10-cent steps for each of the test tones for each group. Pairwise comparisons among the test tones were also performed using a Sidak adjustment. The significant (alpha 0.05) pairwise comparisons from these analyses are additionally shown. The RGD is additionally shown for comparison purposes in Figure 11. Upon visual inspection, all three groups show a pattern of error that is congruent with the idea that individuals' estimates were to some degree variable. Higher pitched items show underestimation, lower pitched items show overestimation and the central item (the mistuned center of the stimulus series) show near zero error.

However, a significant interaction in the omnibus Anova between Target tone and Group [*F*_(18, 270)_ = 3.398, *p* < 0.001] suggests that the pattern of error differed across the groups. One possibility is that while AP possessors' estimates were variable to some degree, their estimates may be still be influenced by note knowledge. For example, it is possible that the point of zero error in individuals with AP may be influenced toward one of the in-tune notes. This is because [B4] and [C5] may differ in familiarity, as C is a much more commonly experienced key signature than B (Simpson and Huron, 1994; Ben-Haim et al., 2014). If this is the case, than it is far more likely for us to see the point of zero error shifted toward C. To test for this, a linear regression line was fitted to each subject's error pattern (and each randomly generated subject's error pattern), which was found by plotting the amount of matching error as a function of the test tone series. From this, the x-intercept was calculated to infer the point of zero error. To determine if any of the groups' x-intercept was significantly different than RGD, the RGD was added as a group. The location of the x-intercept within the test series was used as a dependent variable in a one-way analysis of variance examining the effects of Group (AP, MN, ME, and the RGD. A significant main effect of Group was found [*F*_(3, 44)_ = 7.59, *p* < 0.001] suggesting that at least one of the groups possess a significantly different central tendency location. Indeed, post hoc pairwise comparison testing using a Tukey HSD test showed that AP possessors' central tendency point was significantly different (alpha 0.05) than MN's, ME's, and the RGD's. More specifically, AP possessors' central tendency point was shifted away from the true center of the distribution, and toward [C5]. AP possessors' zero error point was near stimulus 16, while MN's, ME's, and the RGD's zero error point was near stimulus 14, the true center of the tested distribution (See Figure 11).

As previous mentioned, it is possible that differences in note knowledge or musical experience may affect the accuracy of pitch estimates for target tones. While individuals with more note knowledge may produce tone estimates that are inaccurate, they may be able to advantageously use long-term note categories to reduce such effects. If this is the case, AP possessors should display significantly less error across the distribution than MN and ME. However, it is also possible that domain general enhancements in working memory and attention, due to experience with Western music, may help individuals to better remember the isolated tones. If this is the case, then AP possessors and ME should display significantly less error across the distribution than MN. Additionally, it will be important to know how the error of individuals' tone estimates compared to the RGD. In Experiment 1, individuals from the general population, while variable in their estimates, showed significantly less error than the RGD. However, the current experiment used a distribution where only two of target tones differed by 20 cents and included two perfectly in-tune notes (B4 and C5). As such it is possible that some groups may not differ from the RGD in the amount of error they show across the distribution.

In Experiment 1, we argued that experience with Western note knowledge leads to less estimation error compared to random responses. If this is truly the case, we should find in Experiment 2 that differences in prior experience with the Western chromatic scale vary the amount of error in individuals' estimates of isolated tones. In order to determine if prior pitch knowledge affects pitch estimation for isolated tones, the amount of error was plotted against the presented test tone series for each subject. A linear regression line was fitted to each subject's estimation function (and to each simulated subject's estimation function). The steepness of the fitted linear regression line was then used to assess the degree to which items were influenced by the central items of the series as, a steeper fitted regression line would necessarily denote more extreme overestimation of smaller items as well as more extreme underestimation of larger items in the series. As such, the slope corresponding to each subject's fitted regression line was used as a dependent measure in a One-Way ANOVA with Group (AP, MN, ME, and the RGD) as the main factor. Indeed, a significant main effect of Group was found [*F*_(2, 32)_ = 69.82, *p* < 0.001]. *Post-hoc* pairwise comparison testing using a Tukey HSD test however, revealed that AP possessors' and ME's error patterns have a significantly flatter slope than the MN's and the RGD's error pattern. This suggests that domain general enhancements in working memory and attention, due to musical experience, helped AP possessors and ME better remember the isolated tones, and as such, helped to make estimates more accurate. Further, all individuals (AP, ME, and MN) showed significantly more accurate responses across the distribution than the RGD, indicating that all groups possess some long-term pitch knowledge that influences the estimation of isolated tones.

In order to verify that our AP possessors, who self-identified as having AP, did indeed possess the ability to produce an isolated note without the aid of a reference note, we analyzed the mean pitch of individuals' produced notes, comparing them to the objective standard used in Western music (A4 = 440 Hz). The pitch of each production was analyzed in Praat using the Burg algorithm (as reported by Press et al., 1992), and we used for analysis the latest possible window of 1 s where participants held a stable pitch. The reason we used the latest possible window for analysis is because only one participant had extensive vocal training, thus we wanted to allow individuals to adjust their initial vocal utterance to match their internal category standard if necessary. Overall, in addition to being self-identified as possessing AP, AP possessors were remarkably accurate at producing isolated musical notes, as the mean difference between their production and the objective tuning standard was less than half a semitone or 50 cents (*M*: −34.8 cents, *SD*: 30.5 cents, range: 21.9 to −45.5 cents). This is quite remarkable as the smallest distance between any two notes in the Western music scale is one semitone or 100 cents. Further, we never provided participants with feedback as to whether their sung note was correct (nor did we ever provide them with feedback throughout the entire experiment). Thus, all AP participants were well within an acceptable range for accurately producing isolated musical notes without the aid of a reference note. Individuals from the ME and MN did not participate in the pitch production task.

### Discussion

Despite a significant difference in musical experience and explicit note knowledge, the tone estimates of AP possessors, MN and ME had surprisingly similar patterns of error (see Figures 8–10). All three groups showed a point of zero error at or near the center of the tested distribution, suggesting that despite extreme differences in prior musical knowledge, individuals' estimates were still to some extent variable. Specifically, all three groups showed a zero error point associated with the center of the distribution such that lower pitched items of the test tone series were overestimated and higher pitched items of the test tone series were underestimated.

While all subjects were given exposure to the tones in the grouping task that preceded the tone matching task, it is still possible that the tone series was just too novel and as such did not bring prior note knowledge to bear on judgments of these tones. In this sense, it is possible that prior note knowledge might have had a more substantial effect on tone estimates if a more familiar timbre (e.g., piano) note series was used. Previous research indicates that AP possessors are more accurate and faster to identify notes when they are familiar with the timbre (Bahr et al., 2005; Schlemmer et al., 2005). Further Schlemmer (2009) has shown a positive correlation between experience with a particular piece and the ability to spontaneously sing it on key without the aid of a reference tone. Indeed, it is reasonable to assert that the use of prior pitch knowledge in the estimation of notes is likely modulated by the timbre, range and tonality of the notes used.

However, despite the probable novelty of the tone series' timbre, range and tonality, there were still notable differences in the pattern of error across the groups. Both AP possessors and ME had significantly less overall error than MN. This is consistent with the idea that domain general enhancements in working memory and attention, due to musical experience, helped individuals to better remember the isolated tones. It is also possible though that the differences between MN and those with musical expertise (which includes both AP possessors and ME) are not solely due to domain-general enhancements in cognitive processing. Previous work has shown that musicians possess a facility for the processing and memory of musical sounds, and that this facility is accompanied by enhancement and more diverse brain activity (Koelsch et al., 1999; Brattico et al., 2001; Gaab and Schlaug, 2003). Wickens (1973) has speculated that this enhanced and wider spread neural activation is reflective of a robust representational system that supports and improves the encoding of auditory events. As such it is possible that the differences between MN and those with musical expertise (which includes both AP possessors and ME) are not completely due to disparities in working memory capacity but also arise from differences in representational richness that exists between experts and non-experts.

**Figure 12:**
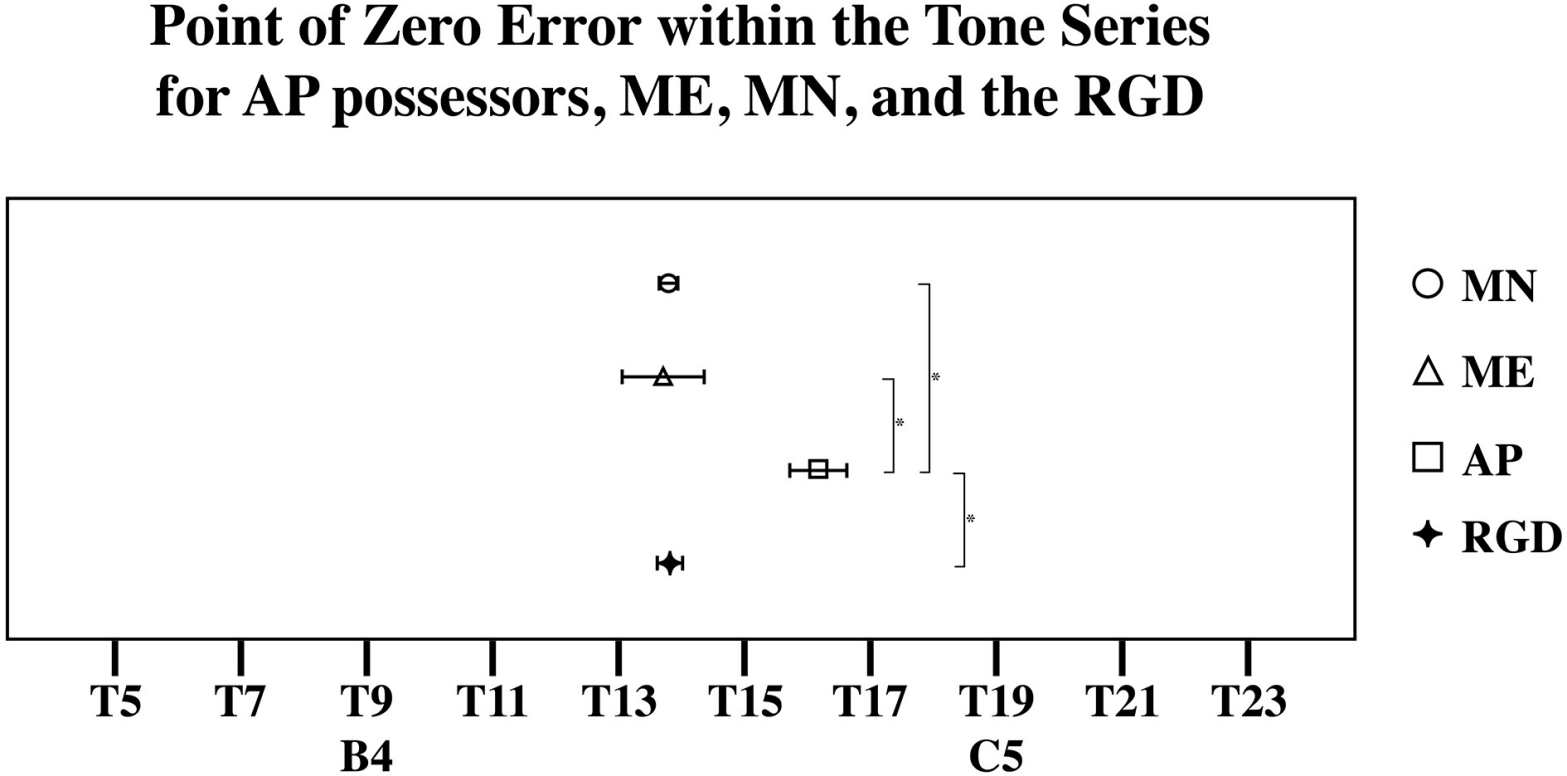
**The location of each group's point of zero error or central tendency point**. Significant pairwise comparisons (alpha 0.05) using a Tukey HSD test are marked by an asterisk [^*^]. Note that AP possessors' central tendency point is shifted away from the true center of the distribution, and toward' [C5].

Beyond demonstrating differences in the amount of variability in individuals' estimates, groups also differed in their point of zero error. More specifically, AP possessors' had a significantly different crossing point, than MN, ME, and the RGD such that AP possessors' point of zero error was near stimulus 16, closer to the in-tune [C5], while MN's, ME's, and the RGD's point of zero error was near stimulus 14, the true center of the tested distribution (See Figure 12). This is commensurate with the notion that prior note category knowledge such as found in AP possessors additionally influenced their estimates of tones. This was demonstrated with a shift in the distributionally based zero point error toward [C5], such that notes closer to [C5] had over all less error. Given that [C5] is a much more common note and key signature than [B4] (Simpson and Huron, 1994; Ben-Haim et al., 2014), the shift toward [C5] in AP subject's estimates provides additional evidence to a growing literature that AP possessors' note representations are based on the statistics of listening experience (Bahr et al., 2005; Schlemmer et al., 2005; Hedger et al., 2013).

## General discussion

Taken together, the results of these experiments provide evidence that musical novices (MN), musical experts (ME) as well as absolute pitch (AP) possessors all possess to some degree prior note knowledge as they all showed less error than the RGD, which is generated only on the basis of stimulus parameters. Further, the amount of prior note experience appears to modulate this error, such that more experience leads to more accurate tone estimates. In addition to these findings, possessing explicit note knowledge, as is the case for those with AP, appears to additionally influenced estimates.

Why would AP possessors, who have absolute pitch knowledge for note categories, demonstrate variability in their estimates that is not systematically related to their note categories? It is perhaps unsurprising that MN or ME make variable estimates, as they do not have explicit absolute pitch knowledge. However, for listeners with rich prior musical note category knowledge such as in AP, the memory of an isolated tone should be structured by long-term note categories. If this was the case, AP listeners should display significantly less error across the stimulus series than ME, who are matched on musical experience. This was not the case, as AP listeners, while showing less matching error than MN, showed similar amounts of matching error to ME. This suggests that the decrease in overall error is not due to robust AP note categories but to domain general enhancements in working memory and attention that stem from extensive musical experience. These results demonstrate that the category knowledge in AP is not as absolute as might be believed. Indeed, Hedger et al. (2013) demonstrated that changing the frequency tuning of notes in a musical piece quickly retunes the note category prototypes for AP listeners to be in accordance with the altered listening experience. Furthermore, this category shift generalized to notes not included in the detuned musical experience (albeit not to a different timbre), suggesting that Absolute Pitch perception is dependent on underlying statistical experience of tone frequencies. It is this generalization beyond the detuned notes experience that demonstrates strongly the systematicity of the note knowledge for AP listeners. The present data similarly suggests that Absolute pitch perception relies on an interaction between category knowledge and stability in listening experience.

From this perspective, the perception of auditory objects might be thought of as an active cognitive process given that perception occurs against the backdrop of prior experience. That is, even when simple tones are presented in isolation, individuals systematically perceive them in the context of prior musical note knowledge to a degree. The more experience one possess, the greater the influence of this knowledge on perception. In this sense, the perception of pitch, even in AP listeners should not be thought of as a simple template matching process. Clearly AP listeners do not directly access a note category from the frequency information in a tone. Rather, pitch information is perceived within the context of previous pitch experience. As such, the active use of prior pitch knowledge in the perception of simple, isolated tones prohibits a model of auditory perception that is simply bottom up. Models of auditory perception should allow for the readjustment of subcortical processing, via the corticofugal system, to engage in egocentric selection, in which input from the brainstem is improved through feedback and lateral inhibition (Suga et al., 2002).

Overall, we have provided empirical evidence that all listeners possess to some degree prior pitch knowledge that affects the perception and subsequent judgments of isolated tones. Moreover, the amount of prior pitch knowledge modulated the degree to which estimates were accurate. Experienced listeners with substantial explicit knowledge and training showed less overall error than listeners without formal explicit training, suggesting that domain general enhancements in working memory and attention are associated with musical experience. Notably, all listeners showed less error than RGD. Additionally, listeners with absolute pitch showed a significant effect of note category knowledge over and above this musical experience. Lastly, the perceptual learning of the intensional structure of note categories does influence the estimates of isolated tones. These data suggest that auditory objects that have intrinsic relationships in pattern structure may be perceived under the influence of prior listening experience. This suggests that the extensional mapping of auditory objects as stimuli onto perceptual experiences follows a common set of principles in common with other psychophysical judgments. However, with sufficient perceptual training and experience, the systematicity of category knowledge can have an effect as well on the perceptual processing of these auditory objects, suggesting an active perceptual processing mechanism to instantiate such category knowledge.

## Author contributions

Shannon L. M. Heald and Stephen C. Van Hedger designed the reported studies and collected data and analyzed the data together with Howard C. Nusbaum. Shannon L. M. Heald prepared the first draft and Stephen C. Van Hedger and Howard C. Nusbaum revised and both refined the manuscript to final form.

### Conflict of interest statement

The authors declare that the research was conducted in the absence of any commercial or financial relationships that could be construed as a potential conflict of interest.
